# California annual grass invaders: the drivers or passengers of change?

**DOI:** 10.1111/j.1365-2745.2010.01706.x

**Published:** 2010-09

**Authors:** Janneke HilleRisLambers, Stephanie G Yelenik, Benjamin P Colman, Jonathan M Levine

**Affiliations:** 1 Biology Department, University of WashingtonSeattle 98195-1800, WA, USA; 2 Ecology, Evolution and Marine Biology, University of CaliforniaSanta Barbara, CA 93106, USA; 3 Department of Forest Ecosystems and Society, Oregon State UniversityCorvallis, OR 97331, USA; 4 Department of Biology, Duke UniversityDurham, NC 27708, USA

**Keywords:** community assembly, competition, conservation, grasslands, grazing, herbivory, invasion ecology, R*

## Abstract

1. The dominance of invasive species is often assumed to reflect their competitive superiority over displaced native species. However, invasive species may be abundant because of their greater tolerance to anthropogenic impacts accompanying their introduction. Thus, invasive species can either be the drivers or passengers of change.
2. We distinguish between these two possibilities in California grasslands currently dominated by Mediterranean annuals (exotics) and subjected to livestock grazing since European settlement. We focused on native annual grasses and forbs, an understudied species-rich component of the California flora, and Mediterranean annual grasses, currently dominant and among the first non-native plants introduced to the area.
3. We established a field experiment with fenced and unfenced blocks in a cattle pasture. We measured concentrations of limiting resources (nitrogen, phosphorus, light and soil moisture) in monoculture plots as an index of competitive ability (i.e. R*). We then quantified grazing impacts on biomass and seed production in grazed vs. ungrazed monoculture plots. Finally, we measured biomass and seed production of each species competing in mixture plots, in the presence and absence of grazers.
4. We found that native and exotic species did not differ in R* indices of competitive ability, i.e. concentrations of limiting resources in ungrazed native monoculture plots did not differ from concentrations in ungrazed exotic monoculture plots. By contrast, exotic annuals suffered less from grazing than native annuals, perhaps reflecting their longer evolutionary history with cattle grazing. Consistent with these results, native and exotic annuals were equally abundant in ungrazed mixtures, but exotic species overwhelmingly dominated grazed mixtures.
5. Species able to draw down nitrogen and light to lower levels in monocultures (i.e. those with lower R* values) dominated biomass and seeds in mixed plots without grazers. However, R* did not predict the relative abundance of species in grazed plots. Moreover, the relative abundance of species in mixtures did not correlate with grazing impacts on their monocultures, implying that grazing alters inter-specific competitive dynamics.
6. *Synthesis*. We demonstrate that the displacement of native annuals by Mediterranean annual grasses in California may largely have been driven by cattle grazing.

## Introduction

Non-native plants can have large negative impacts on the ecosystems they invade. For example, a Eurasian grass (*Bromus tectorum*) has increased fire frequencies in the western US (Knapp 1996), an Australian tree (*Melaleuca quinquenervia*) has changed the hydrology of the Florida everglades (Gordon 1998), and an African tree (*Myrica faya*) has altered nitrogen cycling in Hawai’i (Vitousek & Walker 1989). Through changes in ecosystem processes as well as direct interactions (e.g. competition, predation), non-native species often decrease native diversity and alter species composition. In fact, recent estimates suggest that up to 40% of endangered species are threatened by non-native plants and animals (Pimentel, Zuniga & Morrison 2005). An understanding of the mechanisms underlying the success of invasive species is therefore of both basic and applied interest, offering plant ecologists valuable insights into the historical and contemporary processes that structure plant communities, as well as informing management efforts (Elton 1958; Vitousek, Loope & Stone 1987; D’Antonio & Vitousek 1992; Sax *et al.* 2007).

Given that non-native species introductions are generally accompanied by other anthropogenic changes to the landscape, it is often difficult to determine whether invasive species are the drivers or passengers of observed changes in community composition (MacDougall & Turkington 2005; Lilley & Vellend 2009). If introduced species are better resource competitors than natives, competitive dynamics may drive the displacement of native species by introduced species. In such cases, the introduction of the invasive species can cause a fundamental and potentially long-term change to communities. Alternatively, if other simultaneously introduced large-scale anthropogenic changes alter competitive dynamics to the benefit of introduced species (e.g. livestock grazing –Hayes & Holl 2003; Parker, Burkepile & Hay 2006), the dominance of non-native species may simply reflect the extent of human influence in an area. Whether invaders are drivers or passengers of change represent the opposite ends of a continuum of possibilities; the dominance of invasive species probably depends both on competitive dynamics with natives and on the influence of other anthropogenic factors on such dynamics (van der Wal *et al.* 2008; Best & Arcese 2009). However, understanding where invasions fall along this continuum could help in focusing research on the factors controlling invasion success, and direct effective management of invasive species.

California grasslands are a dramatic example of an invaded landscape, in which the current dominance of Mediterranean annual grasses could reflect their competitive ability or their greater tolerance of anthropogenic factors. Over the course of the last two centuries, California grasslands have been converted to a community dominated by a suite of non-native Mediterranean annuals, primarily grasses (Jackson 1985; Seabloom *et al.* 2003). The dramatic and continued success of these Mediterranean annual grasses (exotics) over native species could indicate that these non-native grasses are on average better competitors for limiting resources, and thus, the drivers of community change. However, the introduction and spread of exotic annual grasses was accompanied by large changes to disturbance regimes, including high-intensity grazing by livestock (Burcham 1956; D’Antonio *et al.* 2007; Jackson & Bartolome 2007). It is thus possible that this new disturbance regime benefited exotic annual grasses over natives (Hayes & Holl 2003), making Mediterranean annual grasses the passengers of anthropogenic land use change.

In the last few decades, much progress has been made in the study of the California grassland invasion by Mediterranean grasses. Several studies suggest that native perennial bunchgrasses, thought to be abundant in pre-invasion grasslands, are not inferior competitors to Mediterranean annual grasses but are often less tolerant of disturbance (Seabloom *et al.* 2003; Corbin & D’Antonio 2004). It is therefore tempting to conclude that Mediterranean annuals are not the drivers of change in California grasslands but abundant because of their association with the wide spread anthropogenic disturbance brought to California by European settlers. However, pre-settlement California grasslands hosted a diverse mixture of native annual forbs and grasses in addition to perennial bunchgrasses, and Mediterranean annual grasses also competed with and displaced these species (D’Antonio *et al.* 2007; Schiffman 2007b). Because the short life cycles of annual plants generally render them less sensitive than perennials to grazing (Diaz *et al.* 2007), the almost exclusive focus of previous studies on interactions between Mediterranean annuals and a handful of native perennial bunchgrasses is problematic (Dyer & Rice 1999; Hamilton, Holzapfel & Mahall 1999; Seabloom *et al.* 2003; Corbin & D’Antonio 2004; but see Hayes & Holl 2003). Why did introduced Mediterranean annuals, rather than pre-existing native annuals, increase so dramatically following the disturbance associated with European settlement? This question motivates our study.

To address this question, we focus on six Mediterranean annual grass species, representing the earliest invaders of California grasslands, and five native annual species (both grasses and forbs), that are widespread in California grasslands today. This group of species allows us to compare successful annual invaders that were among the first to be introduced to California (grasses) with those native annuals that were displaced (forbs and grasses). We experimentally assembled single-species stands and mixed species communities of native and exotic annuals and manipulated grazing with fences. We used monoculture plots to quantify species-specific indices of competitive ability (R*–Tilman 1982) and the impacts of grazing on native and exotic species. Finally, we determined the outcome of competition in mixed species plots, both in the absence and presence of grazing. In combination, these measurements allowed us to ask: (i) whether exotic annual grasses are more competitive than native annuals, and thus, the drivers of change, (ii) whether exotic annual grasses are less impacted by grazing than natives, and thus, the passengers of change, and (iii) whether the relative abundance of exotic and native annuals in mixed plots is explained by competitive dynamics, grazer impacts, or both.

## Materials and methods

### Site

We established the experiment in a cattle pasture in Santa Ynez Valley, Santa Barbara County, California. The climate is Mediterranean, with precipitation primarily falling between November and March. Rainfall averages 521 mm annually, but varies greatly between years (standard error of 37.7, 479 mm in the year of the experiment). August is the warmest month with maximal daytime temperatures of 34 °C, and January is the coldest month with maximal daytime temperatures of 19 °C. Like other grasslands in central and southern California, vegetation consists of a mixture of annual and perennial herbaceous forbs and grasses (primarily exotic annual grasses) growing with occasional oak trees (Coast Live Oak, *Quercus agrifolia* and Valley Oak, *Quercus lobata*). Soils are Typic Argixerolls with a gravelly fine sandy loam texture (Soil Survey Staff, Natural Resources Conservation Service, USDA Web Soil Survey –http://websoilsurvey.nrcs.usda.gov/).

The pasture has been in the possession of Midland School since the 1940s and has never been tilled or ploughed for crops. In 2006 (the year of the experiment), the 500 acre pasture was stocked with ∼150 cow/calf pairs, and the residual dry matter in grazed plots was 46.8 g m^−2^ (standard error 4.14) as compared to 281.2 g m^−2^ in ungrazed plots (standard error 24.8). This is on the high end of grazing intensity currently recommended in California (Bartolome *et al.* 2002), but likely representative of the high grazing pressures present when exotic annual grasses were first introduced to the region (Burcham 1956; D’Antonio *et al.* 2007). Cattle have grazed these lands annually since the 1940s, and possibly for longer. As is common grazing practice in the area (B. Munger, Midland Ranch Manager, pers. comm.), cattle were introduced to the pasture in the spring (late February/early March), a month or two after our study species germinated, and were removed in late summer (July/August), well after peak biomass (when biomass harvests occurred).

### Experimental design

Five blocks were established in the pasture, separated from each other by 50–500 m. Seeds in the seedbank were allowed to germinate following the first rain of the growing season (October 2005), after which extant vegetation was removed with Roundup® (a.i. glyphosate, 0.85% concentration) and two passes by a tractor with a disc harrow (no roller). A rake was then used to level the ground and remove large clumps of vegetation. Each block was then equally divided into halves (separated by roughly 8 m), with a fence built around one half of the block using fence posts every 2 m and four strands of barbed wire. Twelve 0.64 m^2^ plots were established within each block half, one monoculture plot for each of the 11 species and one ‘competition’ plot in which all species were grown together. In total, we established 120 plots, equally divided between the five blocks, between grazed and ungrazed conditions, and replicated by species composition (monoculture plots for each species, a mixture plot with all species).

We initiated the experiment by seeding three native annual forbs, three native annual grasses and six Mediterranean annual grasses into plots in autumn of 2005. Seeds of one native grass (*Muhlenbergia microsperma*) did not germinate, so we do not consider it in the remaining analyses. We focus on Mediterranean grasses, because they were among the earliest non-native species introduced to California grasslands (D’Antonio *et al.* 2007). By contrast, we focus on native annual forbs and grasses, because annual forbs and grasses were thought to be abundant in California grasslands prior to European settlement (Schiffman 2007b). Thus, our comparison probably reflects realistic interactions between some of the earliest non-native species introduced to the region (exotic annual grasses) and the native annual species that were displaced. We chose only species that occurred at or near our study site.

Seeds were collected locally (*Avena barbata*, *Bromus hordeaceus*, *Hordeum murinum*, *Lamarckia aurea*, *Vulpia microstachys* and *Vulpia myuros*) or ordered from a seed company (*Amsinckia menziesii*, *Calandrinia ciliata*, *Clarkia purpurea*, *Polypogon monspeliensis* and *Vulpia octoflora –* provided by S&S seeds, http://www.ssseeds.com/). S&S seeds derived from populations collected in Santa Barbara County and are propagated locally. Thus, genetic or maternal effects are likely to be small. We added 15 g of seed m^−2^ to plots, divided equally among all species in mixture plots. The number of seed added per species in each mixture plot was 1226 on average, but since seed size varies per species, this ranged between 119 seeds (*A. barbata*) and 2822 seeds (*C. ciliata*). Seeds were added in late November and plots were watered with the equivalent of 75 mm of rainfall just after seeding to encourage germination and establishment. Plots were weeded twice, soon after germination and midway through the growing season to remove non-target species. This amount of seed resulted in densely vegetated plots with little bare ground visible in ungrazed plots.

### Measurements

Resource competition theory (developed by Tilman 1982) predicts that the concentration of limiting resources in monocultures (termed R*) is a species-specific measure of resource drawdown, and thus predicts the outcome of competitive dynamics in a resource limited community (lower R* species are assumed to be more competitive). Since the development of this theory, several field studies have verified that R* for limiting resources such as nitrogen and light is often correlated with dominance or the outcome of competition in terrestrial plant communities (e.g. Wedin & Tilman 1993; HilleRisLambers *et al.* 2004; Harpole & Tilman 2006; Vojtech, Turnbull & Hector 2007; Banta *et al.* 2008; Violle *et al.* 2009). We therefore measured R*, resource concentrations in ungrazed monoculture plots, as a species-specific index of competitive ability for our 11 species. Because species were randomly assigned to plots within blocks, we assume that differences in resource concentrations in monoculture plots are related to species-specific differences in resource uptake throughout the growing season. Nitrogen concentrations could also be affected by species-specific impacts on microbial communities and their process rates (e.g. Wedin & Tilman 1990; Hobbie 1992; Van der Krift & Berendse 2001); however, measurements of C : N ratios in plant and microbial biomass, as well as measures of soil N fluxes and pools provide no evidence for such plant-soil feedbacks in this experiment (S. G. Yelenik, unpublished data).

We measured nitrogen, phosphorus and water (soil moisture), and quantified light interception by the canopy in monoculture plots. Measurements were made once, during the height of the growing season, just as species were starting to set seed, and within 2 weeks of above-ground biomass harvests. Phenological differences between species probably influenced these values. However, we assume these effects are small, as phenology was not correlated with R* measurements. We assume that one-time measurements of resource concentrations at peak biomass are an index of the integrated ability of species to draw down these resources during the entire growing season.

We measured soil resources by extracting two soil cores (5 cm diameter, 10 cm depth) from each plot. The two cores were combined and sieved prior to analyses for N, P and soil moisture. Inorganic nitrogen was quantified using a 2 m KCL extraction, and per cent soil moisture was determined gravimetrically after drying a known mass of soil for 6 days at 60 °C. Phosphorus levels were determined using a resin extraction method (Kuo 1996, as modified by D. Turner –http://www.stanford.edu/group/Vitousek/resinp.htm). Resin bags were calibrated with solutions of known concentration, and sample values were corrected according to the linear relationship between standard and extracted values. Soil nitrate, ammonium and phosphorus in extracts were measured using a Lachat 2300 autoanalyzer (Lachat Instruments, Milwaukee, WI, USA). As our measure of resource uptake for nitrogen, we added nitrate and ammonium concentrations to yield dissolved inorganic nitrogen (DIN).

We measured photosynthetically active radiation (PAR) above and below the plant canopy in two locations in each plot, using a 1-m long Decagon light meter. These measurements were made on the same cloudless day between 11 : 00 am and 2 : 00 pm, at peak biomass. We use these two measures to determine the per cent of light reaching the soil surface as our measure of R* for light. We assume that the lower this percentage (i.e. the greater the amount of light intercepted by the plant canopy), the greater the ability of that species to compete for light.

We quantified production and seed production per species in all plots to determine grazing impacts (in monocultures) and relative abundance in competition. We quantified production by clipping all biomass in a 10 × 50 cm area within the plot. In mixture plots, we sorted the biomass into labelled paper bags while clipping. After clipping, biomass was dried in a drying oven (at 60 °C) for 6 days before being weighed to the nearest 0.001 g. We also quantified seed production at the time of seed dispersal for each species, by quantifying inflorescence density (i.e. maturing fruits/pods for forbs) within a 25 × 25 or 10 × 10 cm square, depending on overall abundance. We then collected three inflorescences per species per plot, removed the seeds, and weighed them. Inflorescence density multiplied by the weight of seeds produced per inflorescence gave us our estimate of seed production for each plot. We separated seeds from pods prior to these calculations for *C. purpurea* and *C. ciliata.* Seeds dispersed rapidly for three species, so we used a species (rather than a plot) level average of seeds/infloresence (*Amsinckia mensiezii*, *C. ciliata)*, or multiplied glume numbers/inflorescence by individual seed weights to yield seed mass per inflorescence (*A. barbata*).

### Statistical analyses

We used linear mixed effects models to test whether resource concentrations in exotic species monoculture plots are lower than in native species plots, which would imply greater resource drawdown (and superior competitive ability) of exotics. We performed four such linear mixed effects models, with soil moisture, nitrogen (DIN), per cent of light reaching the soil surface, and phosphorus levels in ungrazed monoculture plots as response variables, and exotic/native status as the fixed explanatory variable. We designated species identity and block as random effects in these models, to account for non-independence of data collected from the same block and species (Crawley 2007). We report the results of analyses on DIN, the sum of inorganic nitrate and ammonium levels, because the two were correlated (*r* = 0.555) and because individual analyses on nitrate and ammonium yielded qualitatively identical results to those on DIN. DIN was log-transformed prior to analyses to fulfil the requirements of normality.

We next determined whether biomass or seed production (both in g m^−2^) of exotic annuals is less affected by grazing than that of native annuals, using linear mixed effects models to account for block and species effects (Crawley 2007). Biomass or seed production in monocultures were response variables in these tests, with exotic/native status, grazing (both categorical) and their interaction as explanatory variables. Both grazing within block and status within species were designated as random effects in these models. Biomass and seed production values were log-transformed prior to analyses to normalize data. If grazing impacts on exotic annuals are less severe than on native annuals, we expected to find a significant interaction between grazing and exotic/native status.

We then asked whether exotics dominate over natives when grown in competition, and whether grazing alters this balance. We applied linear mixed effects models to species-specific biomass or seed mass data, the response variable, from mixture plots after log transformation. To allow log transformation, we substituted half the smallest non-zero value of (species-specific) biomass or seed production observed across all plots for zero values. Categorical explanatory variables were status (exotic/native), grazing, and their interaction; with species and block designated as random effects for status and grazing, accommodating block and species effects (Crawley 2007). If exotic species dominate over native species regardless of grazing, we expected to find a significant negative coefficient for native status in biomass and seed production mixed effects models. If grazing benefits exotic species in mixtures, we expected to find a significant negative interaction between grazing and native status.

Finally, we asked whether the relative abundance of species in mixture plots reflects competitive ability or grazing impacts. Our measures of competitive ability for each species are block averages of concentrations of each resource in monoculture (i.e. R*). Our measures of species-specific grazing impacts are block averages of biomass (or seed mass) produced in grazed plots subtracted from the biomass (or seed mass) produced in ungrazed plots (on a log scale, i.e. grazing impacts). Our estimate of relative abundance for each of the 11 species is species-specific biomass (or seed mass) produced in a mixture plot divided by the total biomass (or seed mass) produced in that plot, averaged over all five blocks. We used Kendall’s tau because grazing impacts and relative abundances were not normally distributed; results were qualitatively similar when using Pearson’s correlation coefficients. We assumed that negative correlations between R* and relative abundance suggest that competitive dynamics are primarily responsible for abundance hierarchies, as more negative R* values indicate greater competitive ability for that resource. By contrast, we assumed that positive correlations between grazing impacts and relative abundance in grazed plots imply that grazing drives abundance hierarchies, as more negative grazing impacts indicate that grazers reduce biomass or seed mass more severely.

All statistical analyses were performed using R version 2.10.1 (R Development Core Team 2009).

## Results

**Fig. 1:**
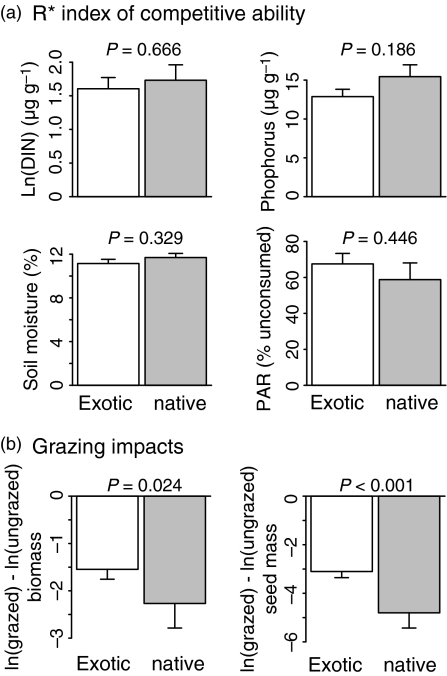
R* for four resources (a) and the impacts of grazing (b) for exotic and native species. R* represents resource concentration in monocultures for DIN (dissolved inorganic nitrogen, nitrate + ammonium concentrations), phosphorus and soil moisture. R* for light (PAR – photosynthetically active radiation unconsumed) is the per cent of light above the canopy that reaches the soil surface. Grazing impacts are the log difference between biomass (or seed mass) in grazed plots and ungrazed plots. Means, standard error bars and *P*-values (in a) are from mixed effects models with exotic/native status as the fixed effect and species and block as random effects. The difference between grazed and ungrazed mass (on a log scale), standard error bars and *P*-values in (b) are from mixed effects models with exotic/native status, grazing, and their interaction as fixed effects and species and block random as effects.

Although the R* index of competitive ability for soil moisture, nitrogen, light and phosphorus varied widely among the 11 species in this experiment (Table 1), exotic species did not differ from native species in R* measurements (Fig. 1a). Specifically, coefficients representing the difference between natives and exotics in resource drawdown were not significantly different from zero for nitrogen concentrations (i.e. DIN; *F* = 0.199, d.f. = 9, *P* = 0.666, Fig. 1a), phosphorus concentrations (*F* = 2.046, d.f. = 9, *P* = 0.186, Fig. 1a), soil moisture (*F* = 1.066, d.f. = 9, *P* = 0.329, Fig. 1a), or light (i.e. PAR; *F* = 0.635, d.f. = 9, *P* = 0.446, Fig. 1a).

**Table 1:** Scientific names, abbreviations, monoculture and mixture biomass productivity, R* indices of competitive ability (one-time measures of resource concentration in monoculture at peak biomass) and grazing impacts on biomass and seeds (the log difference between grazed and ungrazed plots) for the 11 focal species. All values are block averages; standard errors are in parentheses. R* values with different superscripted letters are significant from each other based on Tukey HSD tests (*P* < 0.05)

	Ungrazed biomass (g m^−2^)		R* (resource concentrations in monocultures)				Grazing impacts (ln(grazed) − ln(ungrazed))	
Species (abbreviation)	Mono	Mixture	DIN (μg g^−1^)*	*P* (μg g^−1^)†	H_2_O (%)‡	Light (%)§	Biomass (ln(g))	Seed (ln(g))
Native								
*Amsinckia menziesii* (*Am*)	374.3 (±22.2)	13.22 (±4.07)	10.2 (±4.48)^a,b^	18.07 (±3.84)	12.0 (±1.17)	46.2 (±4.46)^a,b^	−1.764 (±0.45)	−3.615 (±0.47)
*Calandrinia ciliata* (*Cc*)	40.40 (±15.8)	17.13 (±8.83)	6.35 (±2.32)^a,b^	13.59 (±2.65)	11.6 (±0.77)	87.6 (±6.17)^d^	−1.419 (±0.24)	−4.685 (±0.62)
*Clarkia purpurea* (*Cp*)	486.9 (±41.6)	99.24 (±15.5)	3.07 (±0.94)^a^	11.46 (±2.73)	12.5 (±0.40)	41.5 (±3.93)^a^	−3.269 (±0.10)	−5.654 (±0.90)
*Vulpia microstachys* (*Vmi*)	292.7 (±33.9)	35.07 (±10.0)	6.92 (±1.84)^a,b^	14.29 (±1.69)	10.7 (±0.85)	44.3 (±7.85)^a,b^	−2.105 (±0.54)	−5.029 (±0.47)
*Vulpia octoflora* (*Vo*)	204.7 (±21.1)	3.318 (±0.82)	14.8 (±5.64)^b^	19.81 (±2.01)	11.6 (±1.11)	74.2 (±6.56)^c,d^	−2.781 (±0.83)	−5.030 (±0.50)
Exotic								
*Avena barbata* (*Ab*)	511.1 (±185.2)	12.60 (±8.64)	5.31 (±1.14)^a,b^	11.25 (±1.53)	11.2 (±0.71)	60.8 (±7.18)^a–c^	−2.545 (±0.62)	−4.237 (±0.72)
*Bromus hordeaceus* (*Bh*)	343.7 (±109.5)	44.56 (±12.34)	4.20 (±0.41)^a,b^	12.54 (±2.08)	11.7 (±0.53)	46.3 (±1.82)^a,b^	−1.544 (±0.36)	−3.885 (±0.20)
*Hordeum murinum* (*Hm*)	192.8 (±30.3)	5.023 (±1.75)	7.45 (±2.31)^a,b^	12.55 (±2.00)	12.4 (±1.00)	80.4 (±3.37)^c,d^	−1.018 (±0.36)	−1.312 (±0.23)
*Lamarckia aurea* (*La*)	315.0 (±26.3)	6.315 (±2.65)	3.77 (±0.56)^a,b^	12.97 (±2.04)	10.3 (±0.69)	68.4 (±5.99)^b–d^	−2.087 (±0.25)	−3.319 (±0.16)
*Polypogon monspeliensis* (*Pm*)	88.36 (±34.2)	0.509 (±0.51)	14.4 (±5.25)^a,b^	14.31 (±3.26)	11.3 (±1.16)	85.8 (±2.28)^c,d^	−0.360 (±0.38)	−2.184 (±0.44)
*Vulpia myuros* (*Vmy*)	275.6 (±60.8)	61.92 (±20.3)	3.50 (±0.48)^a,b^	13.66 (±1.33)	9.9 (±0.31)	63.4 (±7.39)^a–d^	−1.726 (±0.24)	−3.677 (±0.45)

* R* values for nitrogen, i.e. dissolved inorganic nitrogen (DIN), are based on the sum of nitrate and ammonium concentrations. Species differ significantly in R* measurements of nitrate (*F* = 2.48, d.f. = 10, *P* = 0.02) and DIN (*F* = 2.059, d.f. = 10, *P* = 0.05).

† Species do not differ significantly in R* measurements of *P* (*F* = 1.60, d.f. = 10, *P* = 0.14).

‡ Species do not differ significantly in R* measurements of soil moisture (*F* = 1.87, d.f. = 10, *P* = 0.08).

§ R* for light is calculated by dividing PAR at the soil surface by PAR immediately above the plant canopy, and multiplying this number by 100 (the per cent of light that reaches the soil surface in monoculture); we assume that the lower this value, the more competitive the species is for light. Species differ significantly in R* measurements of light (*F* = 9.88, d.f. = 10, *P* < 0.001).

By contrast, the impacts of grazing on biomass and seed mass in monoculture plots differed between natives and exotics. In the absence of grazing, monoculture plots of exotics did not produce more biomass or seed than native monoculture plots (*F* = 0.675, d.f. = 9, *P* = 0.433 for the difference in log biomass between natives and exotics; *F* = 1.83, d.f. = 9, *P* = 0.209 for the difference in log seed mass between natives and exotics). Both biomass and seed production of natives and exotics were negatively affected by grazing (*F* = 147.52, d.f. = 53, *P* < 0.001 for grazing effects on log biomass; *F* = 431.51, d.f. = 53, *P* < 0.001 for grazing effects on log seed mass). However, exotic annuals were much less negatively impacted than native annuals, resulting in a smaller reduction of biomass or seed mass in grazed vs. ungrazed plots (*F* = 5.412, d.f. = 53, *P* = 0.024 for the interaction between native status and grazing effects on log biomass; *F* = 20.58, d.f. = 53, *P* < 0.001 for the interaction between native status and grazing effects on log seed mass; Fig. 1b).

**Fig. 2:**
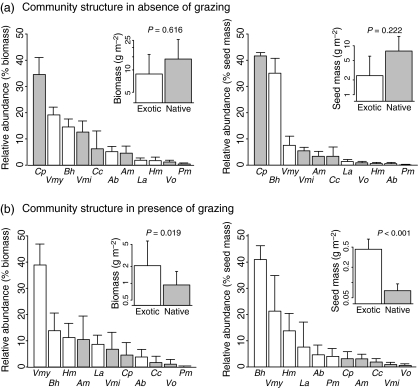
Rank abundance in mixed plots of 11 native and exotic annuals as biomass and seed production in the absence (a) and presence (b) of grazing. Grey bars are native species, white bars are exotic species. Species abbreviations are listed in Table 1. Standard error bars are from five block-specific values of relative abundance (species mass/total mass). Insets show mean biomass and seed production (in g m^−2^) of exotic and native annual species (*y* axis on a log scale). Means, standard error bars and *P*-values in inset graphs are from mixed effects models with exotic/native status, grazing and their interaction as the explanatory variable for biomass or seed production, with species and block as random effects for status and grazing, respectively.

Natives and exotic species were equally abundant in ungrazed mixtures, both as biomass and seed mass (*F* = 0.008, d.f. = 9, *P* = 0.930 for the difference in log biomass between natives and exotics; *F* = 0.006, d.f. = 9, *P* = 0.939 for the difference in log seed mass between natives and exotics; Fig. 2a). In total, native species made up 59% of biomass and 54.8% of seed mass in ungrazed mixture plots. Biomass and seed mass of both natives and exotics decreased in mixtures exposed to grazers (*F* = 60.37, d.f. = 53, *P* < 0.001 for grazing effects on log biomass; *F* = 157.58, d.f. = 53, *P* < 0.001 for grazing effects on log seed mass). However, it decreased more strongly for native species (*F* = 5.85, d.f. = 53, *P* = 0.019 for the interaction between native status and grazing effects on log biomass in mixtures; *F* = 38.38, d.f. = 53, *P* < 0.001 for the interaction between native status and grazing effects on log seed mass in mixture; Fig. 2b). As a result, native species declined to 24.5% of total biomass and 9.71% of total seed mass in grazed mixture plots. These differences were also reflected in the rank abundance of natives and exotics in mixture; in the absence of grazing, common and rare species in mixtures included both native and exotic species (Fig. 2a). By contrast, exotic species were more abundant than natives in terms of biomass with grazers present, and exotics outproduced seed of all native species when grazed (Fig. 2b).

**Fig. 3:**
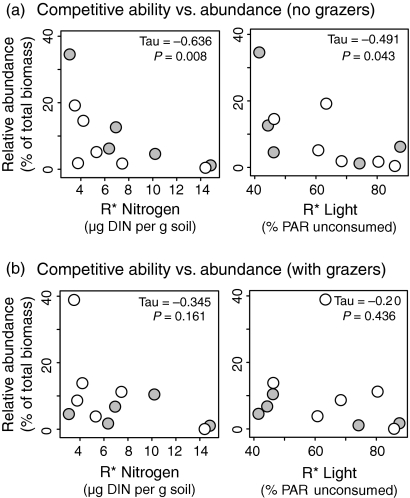
The relationship between competitive ability for nitrogen and light (R*– resource concentrations in monoculture) and relative abundance as biomass in ungrazed (a) and grazed mixtures (b). Lower values of R* indicate greater competitive ability for that resource. Each circle represents one of five native (grey) or six exotic (white) species. *P*-values and Kendall’s τ are based on two-tailed tests; Kendall’s τ is a measure of association between ranked variables. Results are similar for relationships between R* for nitrogen and light and relative abundance in seed mass (results not shown).

When competing in the absence of grazing, the relative abundance of the 11 species as standing biomass was negatively correlated with R* for soil nitrogen (DIN: nitrate + ammonium) and for light (the drawdown of light in their monocultures, Fig. 3a). A similar relationship between relative abundance of the 11 species in total seed mass and R* for nitrogen and light emerged, except that the relationship was only marginally significant for nitrogen (τ = −0.455, *P* = 0.062 for DIN; τ = −0.527, *P* = 0.029 for PAR). In grazed plots, relative abundance in biomass and seed mass was not correlated with R* for nitrogen and light (see Fig. 3b for biomass results; seed mass results: τ = −0.382, *P* = 0.119 for DIN, τ = −0.018, *P* > 0.999 for PAR). Relative abundance in biomass and seed mass in either grazed or ungrazed mixture plots was not significantly correlated with R* for phosphorus or soil moisture (results not shown). In other words, species identified as more competitive for nitrogen and light by R* dominated mixed species plots as biomass and seed after one growing season. In the presence of grazing, however, the relative abundance of the 11 species as biomass or seed was not correlated with R* for light and nitrogen (Fig. 3b). Finally, species that were less impacted by grazing in monocultures (Table 1) did not dominate grazed mixtures as biomass or seed (τ = 0.2, *P* = 0.436 for the relationship between grazing impacts on biomass and relative abundance as biomass; τ = 0.418, *P* = 0.087 for the relationship between grazing impacts on seed and relative abundance as seed).

## Discussion

Mediterranean annual grasses appear to be the passengers, not drivers of the conversion to exotic annual grasslands in California. We found that as a group, exotic annual grasses were not superior to native annuals in their ability to draw down limiting resources, as measured by concentrations of those limiting resources in monocultures (Fig. 1a, Table 1, Tilman 1982; Wedin & Tilman 1993). Studies comparing native perennial grasses to many of the same exotic annual invaders yielded similar conclusions (Seabloom *et al.* 2003; Corbin & D’Antonio 2004). By contrast, grazing strongly favoured exotic grass invaders, driving the natives to produce less seed than any of their exotic counterparts (Figs 1b and 2b). Thus, our results support the hypothesis that exotic annual grasses are more prevalent than native annuals in California grasslands because they were favoured by an intense and long-term anthropogenic disturbance – cattle grazing (Hayes & Holl 2003; Kimball & Schiffman 2003). This is consistent with other studies suggesting that introduced herbivores can promote greater abundance of exotic plant species (Holmgren *et al.* 2000; Parker, Burkepile & Hay 2006; Gonzales & Arcese 2008; Best & Arcese 2009).

Why were native annuals more heavily impacted by grazing than Mediterranean annual grasses (Figs 1b and 2b)? One possibility is that coevolution with humans and their domesticated livestock may give exotic annual grasses from Europe an advantage over native annuals in California (Ricotta *et al.* 2009). When introduced to California, Mediterranean annual grasses had experienced the intense, high-density grazing regimes associated with cattle and other livestock for over 6000 years of their evolutionary history (Perevolotsky & Seligman 1998). By contrast, native annuals in California experienced little persistent grazing by large herbivores since a megafaunal extinction event over 10 000 years ago (Edwards 2007), although they would probably have experienced browsing by elk and pronghorn (Jackson & Bartolome 2007). Studies in other systems have also shown that species sharing a long evolutionary history with herbivores are less negatively impacted by those herbivores than ‘naïve’ native species (Milchunas & Lauenroth 1993; Holmgren *et al.* 2000; Adler *et al.* 2004; Diaz *et al.* 2007). It is interesting to note that this advantage is one that apparently has persisted for more than 150 years after the original introduction of the invaders and livestock (Burcham 1956; Adler *et al.* 2004). Traits allowing species to remain competitive under intense grazing regimes (e.g. growth form, tissue nutrient concentration –Adler *et al.* 2004; Diaz *et al.* 2007) may be slow to evolve in California native annuals, despite many generations of exposure to a strong selective pressure. In addition, native California annuals are predominantly forbs (Schiffman 2007b) while many exotic annuals are grasses. Life form difference between the groups may therefore also explain their differential susceptibility to grazing (Stebbins 1981; Coughenour 1985). Distinguishing between these evolutionary possibilities is beyond the scope of this study.

The large negative impacts of grazing on native annuals could be caused by a differential grazer preference for the native annuals or from their inability to recover from grazing (or both). Our one-time measure of grazing impacts does not allow us to distinguish between these possibilities, although clearly, impacts were greater on natives than exotics (Fig. 1b). The impact of grazing on native seed production was even greater than that on biomass, with exotics making up >75% of biomass in grazed mixtures, but >90% of the seed production (Fig. 2b). Native annual seed production may have been even more sensitive to grazing than biomass production (Del-Val & Crawley 2005). Alternatively, cattle may have targeted the nutrient rich flowering stems or inflorescences of natives (Bazzaz *et al.* 1987; Hülber *et al.* 2005) while avoiding the often spikier seedheads of the exotic grasses (Arnold 1987; Ginane, Petit & D’Hour 2003; Ginane & Petit 2005). Regardless of the relative importance of feeding preference, grazing tolerance or recovery following grazing, the strong impacts on native seed production we observed could have favoured exotic annuals and resulted in rapid compositional changes under the intense and widespread grazing regimes imposed when Mediterranean annuals were introduced to the region (Jackson & Bartolome 2007).

If grazing in California grasslands benefits exotic annual grasses, as suggested by our study (Fig. 2), why is grazing sometimes recommended for management of invasive plant species? (e.g. Collins *et al.* 1998; Weiss 1999; Germano, Rathbun & Saslaw 2001; Marty 2005; Huntsinger, Bartolome & D’Antonio 2007). One reason may be that the loss of keystone herbivores often results in declining plant diversity or increased abundance of invasive species, implying that herbivores can benefit native plants (Hobbs & Huenneke 1992; Collins *et al.* 1998, Ripple & Beschta 2006; van der Wal *et al.* 2008). Additionally, several studies in California have documented an increase in invasive species abundance following the removal of grazers (Weiss 1999; Marty 2005). These two studies differed from ours by focusing on interactions between exotic annual grasses and the short-statured native species that occur in more specialized edaphic conditions (serpentine outcrops and ephemeral wetlands). Our study, by contrast, explored the effects of a high-intensity grazing regime (as probably occurred with European settlement) on native plants that can potentially compete with exotic annuals in the absence of grazing (Fig. 1a). It is possible that intermediate levels of grazing, or a narrower window of grazing relative to plant phenological stages, would not have such negative impacts on native California annuals. Grazing impacts on native diversity probably depend on context and require further study (Stohlgren, Schell & Vanden Heuvel 1999).

We found it surprising that the exotic annual grasses were not superior competitors for limiting resources compared to native annuals, as measured by R* (Fig. 1a). It is unlikely that we missed measurement of a critical limiting resource, as recent manipulative studies in nearby grasslands and a meta-analysis of resource addition experiments suggest that the resources we examined (light, nitrogen, phosphorus and water) are indeed limiting in California grasslands (Harpole, Goldstein & Aicher 2007; Going, HilleRisLambers & Levine 2009). Moreover, the dominance of low R* species in ungrazed mixtures (Fig. 3) implies that the R* index of competitive ability (resource concentrations in monoculture) reasonably predicted the outcome of competition in this grassland, despite common criticisms of this approach (e.g. Craine, Fargione & Sugita 2005). It is, of course, possible that we would have identified exotic annuals as superior competitors in different sites or different years. For example, Hobbs, Yates & Mooney (2007) found that an exotic annual grass increased in abundance in years with high rainfall. However, two recent studies on the competitive interactions between exotic annual grasses and native perennial grasses also found that exotic annuals are not superior resource competitors, further suggesting that competitive interactions are probably not solely responsible for the overwhelming dominance of exotic annual grasses in California grasslands (Seabloom *et al.* 2003; Corbin & D’Antonio 2004; but see Dyer & Rice 1999). This is not to say that competitive dynamics are not important in this system. Abundance hierarchies of annual species in this experiment were correlated with the R* index of competitive ability in the absence of grazing (Fig. 3). Moreover, grazing impacts on species growing in monocultures could not explain their relative abundance in grazed mixtures, suggesting that competitive dynamics are altered, rather than absent when grazers are present (as in Mulder & Ruess 1998; Stohlgren, Schell & Vanden Heuvel 1999; Van Der Wal *et al.* 2000; Kuijper, Nijhoff & Bakker 2004).

This study cannot unequivocally identify all factors that contributed to the conversion of California grasslands to their current non-native dominated state. It is likely that there are several drivers of exotic species as dominants. For example, other studies have identified differences in seed production and emergence, altered plant–pathogen relationships and burrowing animals as contributing to the dominance of Mediterranean annuals in these systems (Hobbs & Mooney 1985, 1995; Malmstrom *et al.* 2005; Borer *et al.* 2007; D’Antonio *et al.* 2007; DiVittorio, Corbin & D’Antonio 2007; Schiffman 2007a; Abraham, Corbin & D’Antonio 2009). Unfortunately, our understanding of the species composition and disturbance regime of these grasslands prior to and immediately after European settlement is poor, complicating inference (Burcham 1956; D’Antonio *et al.* 2007; Edwards 2007; Huntsinger, Bartolome & D’Antonio 2007; Schiffman 2007b). Nevertheless, our results are consistent with grazing playing a significant role in the displacement of these native annuals by Mediterranean annual grasses (Figs 1b and 2b, Hayes & Holl 2003; Kimball & Schiffman 2003).

What does the future hold for California grasslands? Our results imply that exotic annual grasses will continue to dominate the grassland we studied under the high levels of grazing currently in place (Fig. 2b, Hayes & Holl 2003). However, exotic annual grasses frequently remain dominant in California after disturbances such as grazing are removed, with native species showing little recovery even decades later (Stromberg & Griffin 1996; Stylinski & Allen 1999; Keeley, Lubin & Fotheringham 2003). This suggests it could take native species a long time to increase from low densities following grazing cessation, perhaps due to seed limitation (Seabloom *et al.* 2003) or adverse interactions at seed and seedling stages (DiVittorio, Corbin & D’Antonio 2007). Moreover, our results suggest that the complete elimination of Mediterranean annual grasses from these grasslands is unlikely, as exotic annuals were as competitive for limiting resources as native species (Fig. 1a). However, our results suggest strongly that the abundance of native California annuals in this grassland could more than double in the long-term by decreasing grazing pressures (Fig 2). Thus, our study adds to the growing body of literature (e.g. Holmgren *et al*. 2000, van der Wal *et al.* 2008; Best & Arcese 2009) suggesting that the elimination of anthropogenic factors that favoured exotic species upon their introduction holds great promise for long-term restoration efforts when non-native species are the passengers of human-mediated disturbance, rather than the drivers of community change.

## References

[b1] Abraham JK, Corbin JD, D’Antonio CM (2009). California native and exotic perennial grasses differ in their response to soil nitrogen, exotic annual grass density, and order of emergence. Plant Ecology.

[b2] Adler PB, Milchunas DG, Lauenroth WK, Sala OE, Burke IC (2004). Functional traits of graminoids in semi-arid steppes: a test of grazing histories. Journal of Applied Ecology.

[b3] Arnold GW (1987). Influence of the biomass, botanical composition and sward height of annual pastures on foraging behavior by sheep. Journal of Applied Ecology.

[b4] Banta JA, Stark SC, Stevens MHH, Pendergast TH, Baumert A, Carson WP (2008). Light reduction predicts widespread patterns of dominance between asters and goldenrods. Plant Ecology.

[b5] Bartolome JW, Frost WM, McDougald NK, Connor M (2002). California guidelines for residual dry matter (RDM) management on coastal and foothill annual rangelands. Agriculture and Natural Resources Publication.

[b6] Bazzaz FA, Chiariello NR, Coley PD, Pitelka LF (1987). Allocating resources to reproduction and defense. BioScience.

[b7] Best RJ, Arcese P (2009). Exotic herbivores directly facilitate the exotic grasses they graze: mechanisms for an unexpected positive feedback between invaders. Oecologia.

[b8] Borer ET, Hosseini PR, Seabloom EW, Dobson AP (2007). Pathogen-induced reversal of native dominance in a grassland community. Proceedings of the National Academy of Sciences of the United States of America.

[b9] Burcham LT (1956). Historical backgrounds of range land use in California. Journal of Range Management.

[b10] Collins SL, Knapp AK, Briggs JM, Blair JM, Steinauer EM (1998). Modulation of diversity by grazing and mowing in native tallgrass prairie. Science.

[b11] Corbin JD, D’Antonio CM (2004). Competition between native perennial and exotic annual grasses: implications for an historical invasion. Ecology.

[b12] Coughenour MB (1985). Graminoid responses to grazing by large herbivores – adaptations, exaptations, and interacting processes. Annals of the Missouri Botanical Garden.

[b13] Craine JM, Fargione J, Sugita S (2005). Supply pre-emption, not concentration reduction, is the mechanism of competition for nutrients. New Phytologist.

[b14] Crawley MJ (2007). The R Book.

[b15] D’Antonio CM, Vitousek PM (1992). Biological invasions by exotic grasses, the grass fire cycle, and global change. Annual Review of Ecology and Systematics.

[b16] D’Antonio CM, Malmstrom C, Reynolds SA, Gerlach J, Stromberg MR, Corbin JD, D’Antonio CM (2007). Ecology of invasive non-native species in California grassland. California Grasslands.

[b17] Del-Val EK, Crawley MJ (2005). Are grazing increaser species better tolerators than decreasers? an experimental assessment of defoliation tolerance in eight British grassland species. Journal of Ecology.

[b18] Diaz S, Lavorel S, McIntyre S, Falczuk V, Casanoves F, Milchunas DG (2007). Plant trait responses to grazing – a global synthesis. Global Change Biology.

[b19] DiVittorio CT, Corbin JD, D’Antonio CM (2007). Spatial and temporal patterns of seed dispersal: an important determinant of grassland invasion. Ecological Applications.

[b20] Dyer AR, Rice KJ (1999). Effects of competition on resource availability and growth of a California bunchgrass. Ecology.

[b21] Edwards SW, Stromberg MR, Corbin JD, D’Antonio CM (2007). Rancholabrean mammals of California and their relevance for understanding modern plant ecology. California Grasslands.

[b22] Elton CS (1958). The Ecology of Invasions by Animals and Plants.

[b23] Germano DJ, Rathbun GB, Saslaw LR (2001). Managing exotic grasses and conserving declining species. Wildlife Society Bulletin.

[b24] Ginane C, Petit M (2005). Constraining the time available to graze reinforces heifers’ preference for sward of high quality despite low availability. Applied Animal Behaviour Science.

[b25] Ginane C, Petit M, D’Hour P (2003). How do grazing heifers choose between maturing reproductive and tall or short vegetative swards?. Applied Animal Behaviour Science.

[b26] Going BM, Hille Ris Lambers J, Levine JM (2009). Abiotic and biotic constraints on the growth and reproduction of three invasive annual grasses on serpentine outcrops. Oecologia.

[b27] Gonzales EK, Arcese P (2008). Herbivory more limiting than competition on early and established native plants in an invaded meadow. Ecology.

[b28] Gordon DR (1998). Effects of invasive, non-indigenous plant species on ecosystem processes: lessons from Florida. Ecological Applications.

[b29] Hamilton JG, Holzapfel C, Mahall BE (1999). Coexistence and interference between a native perennial grass and non-native annual grasses in California. Oecologia.

[b30] Harpole WS, Goldstein L, Aicher R, D’Antonio CD, Corbin J, Stromberg M (2007). Resource limitation. California Grasslands.

[b31] Harpole WS, Tilman D (2006). Non-neutral patterns of species abundance in grassland communities. Ecology Letters.

[b32] Hayes GF, Holl KD (2003). Cattle grazing impacts on annual forbs and vegetation composition of mesic grasslands in California. Conservation Biology.

[b33] HilleRisLambers J, Harpole WS, Tilman D, Knops J, Reich PB (2004). Mechanisms responsible for the positive diversity-productivity relationship in minnesota grasslands. Ecology Letters.

[b34] Hobbie SE (1992). Effects of plant-species on nutrient cycling. Trends in Ecology & Evolution.

[b35] Hobbs RJ, Huenneke LF (1992). Disturbance, diversity, and invasion – implications for conservations. Conservation Biology.

[b36] Hobbs RJ, Mooney HA (1985). Community and population-dynamics of serpentine grassland annuals in relation to gopher disturbance. Oecologia.

[b37] Hobbs RJ, Mooney HA (1995). Spatial and temporal variability in California annual grassland – results from a long-term study. Journal of Vegetation Science.

[b38] Hobbs RJ, Yates S, Mooney HA (2007). Long-term data reveal complex dynamics in grassland in relation to climate and disturbance. Ecological Monographs.

[b39] Holmgren M, Aviles R, Sierralta L, Segura AM, Fuentes ER (2000). Why have European herbs so successfully invaded the Chilean matorral? effects of herbivory,soil nutrients, and fire. Journal of Arid Environments.

[b40] Hülber K, Ertl S, Gottfried M, Reiter K, Grabherr G (2005). Gourmets or gourmands? diet selection by large ungulates in high-alpine plant communities and possible impacts on plant propagation. Basic and Applied Ecology.

[b41] Huntsinger L, Bartolome JW, D’Antonio CM, Stromberg MR, Corbin JD, D’Antonio CM (2007). Grazing management on California’s mediterranean grasslands. California Grasslands.

[b42] Jackson LE (1985). Ecological origins of Californias Mediterranean grasses. Journal of Biogeography.

[b43] Jackson RD, Bartolome JW, Stromberg MR, Corbin JD, D’Antonio CM (2007). Grazing ecology of California grasslands. California Grasslands.

[b44] Keeley JE, Lubin D, Fotheringham CJ (2003). Fire and grazing impacts on plant diversity and alien plant invasions in the southern sierra nevada. Ecological Applications.

[b45] Kimball S, Schiffman PM (2003). Differing effects of cattle grazing on native and alien plants. Conservation Biology.

[b46] Knapp PA (1996). Cheatgrass (*Bromus tectorum* L) dominance in the great basin desert – history, persistence, and influences to human activities. Global Environmental Change-Human and Policy Dimensions.

[b47] Kuijper DPJ, Nijhoff DJ, Bakker JP (2004). Herbivory and competition slow down invasion of a tall grass along a productivity gradient. Oecologia.

[b48] Kuo S, Sparks DL (1996). Phosphorus. Methods of Soil Analysis Part III.

[b49] Lilley PL, Vellend M (2009). Negative native-exotic diversity relationship in oak savannas explained by human influence and climate. Oikos.

[b50] MacDougall AS, Turkington R (2005). Are invasive species the drivers or passengers of change in degraded ecosystems?. Ecology.

[b51] Malmstrom CM, McCullough AJ, Johnson HA, Newton LA, Borer ET (2005). Invasive annual grasses indirectly increase virus incidence in California native perennial bunchgrasses. Oecologia.

[b52] Marty JT (2005). Effects of cattle grazing on diversity in ephemeral wetlands. Conservation Biology.

[b53] Milchunas DG, Lauenroth WK (1993). Quantitative effects of grazing on vegetation and soils over a global range of environments. Ecological Monographs.

[b54] Mulder CPH, Ruess RW (1998). Effects of herbivory on arrowgrass: interactions between geese, neighboring plants, and abiotic factors. Ecological Monographs.

[b55] Parker JD, Burkepile DE, Hay ME (2006). Opposing effects of native and exotic herbivores on plant invasions. Science.

[b56] Perevolotsky A, Seligman NG (1998). Role of grazing in Mediterranean rangeland ecosystems – inversion of a paradigm. BioScience.

[b57] Pimentel D, Zuniga R, Morrison D (2005). Update on the environmental and economic costs associated with alien-invasive species in the united states. Ecological Economics.

[b58] R Development Core Team (2009). R: A Language and Environment for Statistical Computing.

[b59] Ricotta C, La Sorte FA, Pysek P, Rapson GL, Celesti-Grapow L, Thompson K (2009). Phyloecology of urban alien floras. Journal of Ecology.

[b60] Ripple WJ, Beschta RL (2006). Linking a cougar decline, trophic cascade, and catastrophic regime shift in Zion national park. Biological Conservation.

[b61] Sax DF, Stachowicz JJ, Brown JH, Bruno JF, Dawson MN, Gaines SD (2007). Ecological and evolutionary insights from species invasions. Trends in Ecology & Evolution.

[b62] Schiffman PM, Stromberg MR, Corbin JD, D’Antonio CM (2007a). Ecology of native animals in California grasslands. California Grasslands.

[b63] Schiffman PM, Stromber MR, Corbin JD, D’Antonia CM (2007b). Species composition at the time of first European settlement. California Grasslands.

[b64] Seabloom EW, Harpole WS, Reichman OJ, Tilman D (2003). Invasion, competitive dominance, and resource use by exotic and native California grassland species. Proceedings of the National Academy of Sciences of the United States of America.

[b65] Stebbins GL (1981). Coevolution of grasses and herbivores. Annals of the Missouri Botanical Garden.

[b66] Stohlgren TJ, Schell LD, Vanden Heuvel B (1999). How grazing and soil quality affect native and exotic plant diversity in rocky mountain grasslands. Ecological Applications.

[b67] Stromberg MR, Griffin JR (1996). Long-term patterns in coastal California grasslands in relation to cultivation, gophers, and grazing. Ecological Applications.

[b68] Stylinski CD, Allen EB (1999). Lack of native species recovery following severe exotic disturbance in southern Californian shrublands. Journal of Applied Ecology.

[b69] Tilman D (1982). Resource Competition and Community Structure.

[b70] Van der Krift TAJ, Berendse F (2001). The effect of plant species on soil nitrogen mineralization. Journal of Ecology.

[b71] Van Der Wal R, Egas M, Van Der Veen A, Bakker J (2000). Effects of resource competition and herbivory on plant performance along a natural productivity gradient. Journal of Ecology.

[b72] Violle C, Garnier E, Lecoeur J, Roumet C, Podeur C, Blanchard A (2009). Competition, traits and resource depletion in plant communities. Oecologia.

[b73] Vitousek PM, Loope LL, Stone CP (1987). Introduced species in Hawai’i – biological effects and opportunities for ecological research. Trends in Ecology & Evolution.

[b74] Vitousek PM, Walker LR (1989). Biological invasion by *Myrica faya* in Hawai’i – plant demography, nitrogen-fixation, ecosystem effects. Ecological Monographs.

[b75] Vojtech E, Turnbull LA, Hector A (2007). Differences in light interception in grass monocultures predict short-term competitive outcomes under productive conditions. PLoS ONE.

[b76] van der Wal R, Truscott A, Pearce ISK, Cole L, Harris MP, Wanless S (2008). Multiple anthropogenic changes cause biodiversity loss through plant invasion. Global Change Biology.

[b77] Wedin DA, Tilman D (1990). Species effects on nitrogen cycling – a test with perennial grasses. Oecologia.

[b78] Wedin D, Tilman GD (1993). Competition among grasses along a nitrogen gradient – initial conditions and mechanisms of competition. Ecological Monographs.

[b79] Weiss SB (1999). Cars, cows, and checkerspot butterflies: nitrogen deposition and management of nutrient-poor grasslands for a threatened species. Conservation Biology.

